# Transcriptional regulation of subclass 5b fimbriae

**DOI:** 10.1186/1471-2180-8-180

**Published:** 2008-10-14

**Authors:** Maria D Bodero, Elizabeth A Harden, George P Munson

**Affiliations:** 1 Department of Microbiology and Immunology, University of Miami Miller School of Medicine, PO Box 016960 (R138), Miami, FL, 33101 USA

## Abstract

**Background:**

Enterotoxigenic *Escherichia coli *(ETEC) is a major cause of infant and child mortality in developing countries. This enteric pathogen causes profuse watery diarrhea by elaborating one or more enterotoxins that intoxicate eukaryotic cells and ultimately leads to a loss of water to the intestinal lumen. Virulence is also dependent upon fimbrial adhesins that facilitate colonization of the small intestine.

**Results:**

The expression of CS1 fimbriae is positively regulated by Rns, a member of the AraC/XylS superfamily of transcriptional regulators. Based on fimbrial protein homology, CS1 fimbriae have been categorized as subclass 5b along with CS17, CS19, and PCFO71 fimbriae. In this study we show that Rns positively regulates the expression of these other subclass 5b members. DNase I footprinting revealed a Rns binding site adjacent to the -35 hexamer of each fimbrial promoter. The CS17 and PCFO71 fimbrial promoters carry a second Rns binding site centered at -109.5, relative to the Rns-dependent transcription start site. This second binding site is centered at -108.5 for the CS19 promoter. Mutagenesis of either site reduced Rns-dependent transcription from each promoter indicating that the molecules bound to these sites apparently function independently of one another, with each having an additive effect upon fimbrial promoter activation.

**Conclusion:**

This study demonstrates that the ETEC virulence regulator Rns is required for the expression of all known 5b fimbriae. Since Rns is also known to control the expression of additional ETEC fimbriae, including those within subclasses 5a and 5c, the inactivation or inhibition of Rns could be an effective strategy to prevent ETEC infections.

## Background

In 2005 diarrheal diseases caused an estimated 1.7 million deaths predominately within low to middle income countries [1]. Approximately 90% of these deaths occur within the first four years of life. Although the burden of diarrheal diseases may decrease within some countries if their economic conditions improve, for citizens of low income nations diarrheal diseases are projected to remain among the ten leading causes of death through 2030 [2]. Although diarrheal disease can be caused by any one of several bacterial or viral pathogens, enterotoxigenic *Escherichia coli *(ETEC) is one of the most frequent causes of diarrhea in developing nations [3-5]. This pathogen causes profuse watery diarrhea by elaborating one or more enterotoxins. Pathogenicity is also dependent upon the expression of fimbriae, which function as adherence factors, allowing the pathogen to colonize the small intestine. To date, over twenty distinct fimbriae have been identified although some of these are more common in affected areas than others [6]. Nevertheless, the diversity of ETEC fimbriae is a significant challenge for the development of vaccines based on fimbrial antigens [7].

Some of the more frequently encountered fimbriae, such as coli surface antigen 1 (CS1), have been characterized in considerable detail. CS1 fimbria are composed of four proteins. These proteins have amino-terminal signal peptides that facilitate their transport to the periplasm via the Sec-dependent general secretory pathway. CooC [GenBank:Q47114] is an outer membrane usher protein that serves as an assembly site and eventually, the base of an assembled CS1 fimbria [8]. In terms of stoichiometry, CooD [GenBank:Q47115] is a minor component localized to the tip of CS1 fimbria while CooA [GenBank:Q6JAY9] polymerizes beneath CooD to form most of the fimbrial structure [9,10]. Although CooB [GenBank:Q6JAZ0] is not found in the final fimbrial structure, it is essential for assembly because it is a chaperone for CooA and CooD, protecting them from proteolysis in the periplasm [11]. CS1 and related fimbriae have been categorized as class 5 fimbriae [12], the alternate chaperone/usher family [13], or more recently α-fimbriae [14]. The latter classification system is based on phylogenetic relationships of fimbrial usher proteins and has the potential to provide a unified and comprehensible system for the classification of fimbriae [14]. Although this new classification system may eventually gain widespread adoption, in the interim we will retain the class 5 nomenclature.

The CS1 operon, *cooBACD*, is transcribed as a polycistronic message from a single promoter (CS1*p*). This operon is carried by the virulence plasmid pCoo [GenBank:CR942285] of ETEC strain C921b-1 or pETEC_73 [GenBank:CP000797] of E24377a. The CS1 promoter is positively regulated by Rns [GenBank:P16114], a member of the AraC/XylS superfamily of regulators [15,16]. In strains C921b-1 and E24377a, Rns is encoded on a virulence plasmid separate from the plasmid carrying the CS1 operon. Rns has two binding sites upstream of *cooB*, one adjacent to the -35 hexamer of CS1*p *and a more distal upstream site [17]. Mutagenesis of either binding site reduces Rns-dependent expression from CS1*p *in vivo.

The CS1 fimbriae are more closely related, by phylogenetic analysis, to CS17 [GenBank:AY515609, GenBank:AY216491], CS19 [GenBank:AY288101], and putative colonization factor O71 (PCFO71) [GenBank:AY513487] fimbriae than other class 5 fimbriae [14,18]. To reflect their relatedness, this group has been designated as subclass 5b [18]. In addition to their similarity based upon protein homology, we show in this study that subclass 5b fimbriae are also similarly regulated by Rns.

## Results

### Transcriptional regulation of subclass 5b fimbrial promoters

Rns is known to regulate one member, CS1, of fimbrial subclass 5b. In addition, the expression of the related CS19 fimbrial proteins is enhanced when CS19+ strains are transformed with a plasmid expressing CfaD [GenBank:P25393] which is 97% identical to Rns [18,19]. However, it is not known if the enhancement of CS19 expression results from positive regulation of the fimbrial promoter or an indirect effect involving non-fimbrial genes within the Rns/CfaD regulon [20,21]. Currently, there is no evidence to suggest that the other subclass 5b members, CS17 and PCFO71, are regulated by Rns. However, the available data does not exclude this possibility. To determine if other subclass 5b fimbria are regulated by Rns, we cloned the promoters of CS17, CS19, and PCFO71 into a promoterless Lac reporter plasmid that was then integrated into the chromosomal *attB*_HK022 _site of K-12 strain MC4100 by site specific recombination. Quantitative enzymatic assays revealed that the reporter strains expressed 8 to 10 times more β-galactosidase when they were transformed with a Rns expression plasmid, pGPMRns, than when they were transformed with the *rns::kan *negative control plasmid pGPMRns<Tn>2 (Table 1). Thus, when these results are combined with previous analyses of the CS1 fimbrial promoter [15], they reveal that all known subclass 5b fimbrial promoters are activated by Rns.

**Table 1:** Activation of fimbrial promoters by Rns

	Mean β-galactosidase activity (Miller Units) ± SD^a^		
Reporter	*rns::kan*	*rns*+	fold activation

CS17*p::lacZYA*	89 ± 3	718 ± 163	8
CS19*p::lacZYA*	487 ± 94	4670 ± 176	10
PCFO71*p::lacZYA*	144 ± 32	1462 ± 139	10

Reporter strains were grown aerobically at 37°C in LB medium with 100 μg/ml ampicillin and harvested from the late log phase cultures with an optical absorbance of 0.4 to 0.6 at 580 nm. ^a^SD, standard deviation; *n *≥ 3.

### Rns-dependent transcription start site

**Figure 1:**
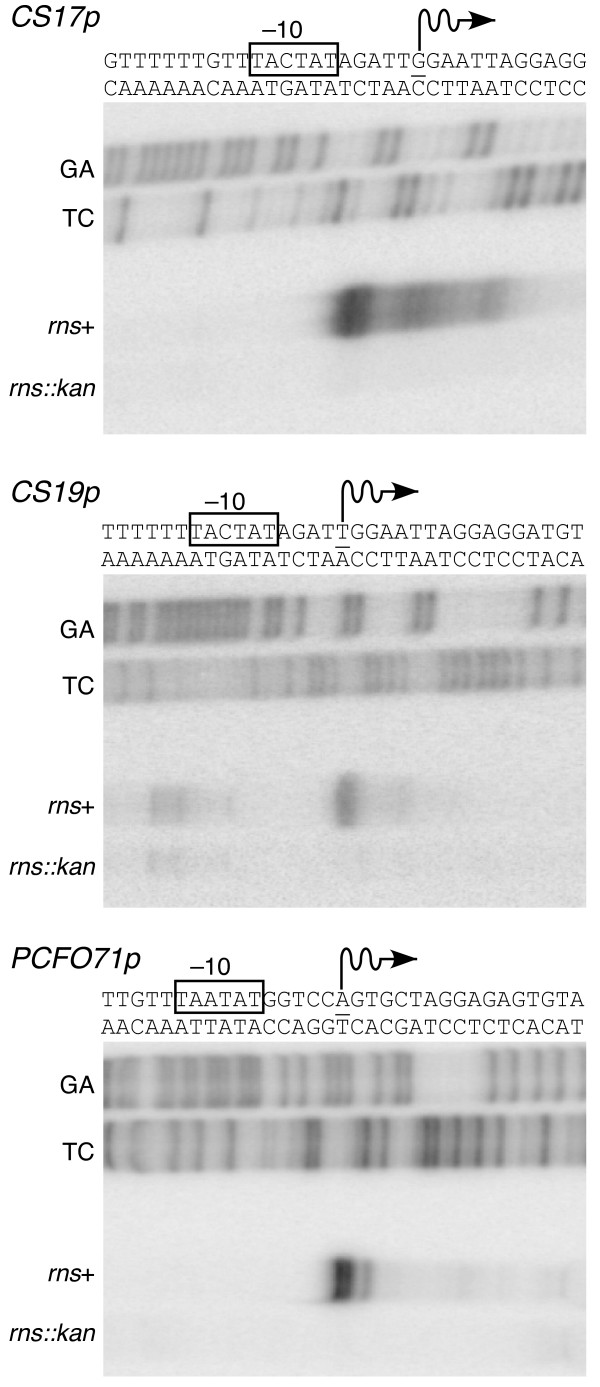
**Rns–dependent transcription start sites of fimbrial promoters**. The transcription start sites of CS17, CS19, and PCFO71 fimbrial promoters were mapped by primer extension of RNA isolated from *rns*+ and *rns::kan *strains. The first nucleotide of each mRNA is underlined and wavy arrows indicate the direction of transcription. Putative–10 hexamers are shown in boxes. Lanes labeled GA and TC contain Maxam–Gilbert sequencing ladders. These are excision reactions and are therefore offset from the primer extension products by -1 nucleotide. RNA was isolated after cultures reached an optical absorbance of 1.0 at 595 nm.

The CS17, CS19, and PCFO71 promoters have not been previously characterized; therefore, the transcription start site of each promoter was mapped by primer extension. We found that the Rns-dependent transcription start site is 16 nucleotides upstream of *csbB *(CS17), 17 nucleotides upstream of *csdB *(CS19), and 20 nucleotides upstream of *cosB *(PCFO71) (Figure 1). We did not observe primer extension products in the absence of Rns although our β-galactosidase assays indicated that each promoter has at least a low level of Rns-independent activity (Table 1). This difference is most likely a consequence of the relatively long half-life of β-galactosidase compared to the short half-life of most mRNA's in *E. coli*. Thus, β-galactosidase assays reflect the accumulative activity of the promoter over an extended period of time while mRNA levels more accurately reflect the promoter's activity at a specific time. Nevertheless, the two different assays, one qualitative the other quantitative, both demonstrate that the CS17, CS19, and PCFO71 fimbrial promoters are positively regulated by Rns.

### Rns binding site position and function

**Figure 2:**
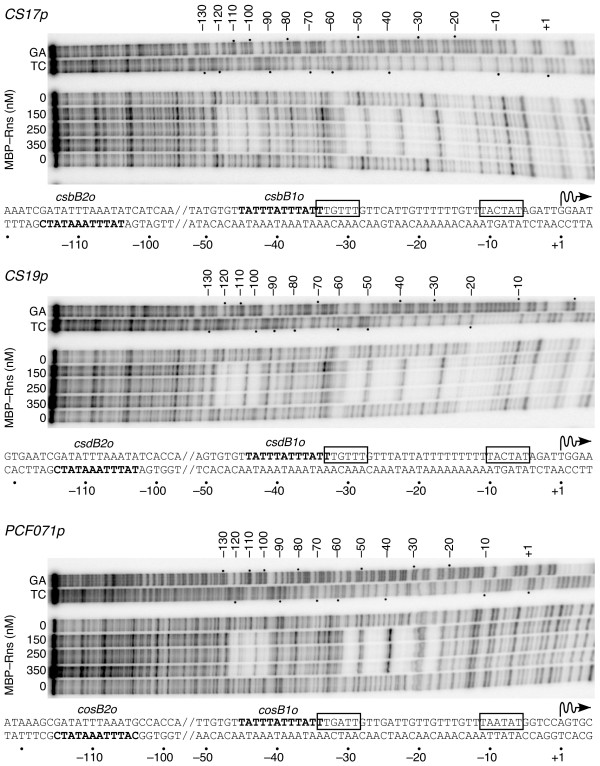
**Identification of Rns binding sites in vitro**. DNase I footprints of MBP–Rns bound the noncoding strands of CS17, CS19, and PCFO71 fimbrial promoters. The nucleotide sequence of each promoter is shown below each gel image with predicted -10 and -35 hexamers in boxes. Numbering is relative to the Rns–dependent transcription start site, denoted by wavy arrows, of each promoter. The central 12 nucleotides of each binding site, which are partially conserved, are shown in bold. Lanes labeled TC and GA contain Maxam–Gilbert sequencing ladders.

In vitro DNase I footprinting was used to determine Rns binding site locations at the CS17, CS19, and PCFO71 promoters. Like other AraC/XylS family members that have been characterized, Rns is too insoluble for the characterization of protein-DNA complexes in vitro. However, it has been previously shown that the fusion of maltose binding protein (MBP) to the amino-terminus of Rns substantially increases its solubility without affecting its activity in vivo [17]. With purified soluble fusion protein we found that Rns binds to a site adjacent to each promoter's -35 hexamer, which were predicted based on primer extension results and sequence analyses (Figure 2). Each promoter also carries a second binding site centered, relative to the Rns-dependent transcription start site, at -109.5 for CS17*p *and PCF071*p *or -108.5 for CS19*p*. DNase I footprints of MBP-Rns typically cover 33 to 36 nucleotides. Due to steric occlusion, these footprints overestimate the actual site of protein-nucleotide contacts [17]. However, each footprint encompasses a central core of 12 nucleotides with at least 67% identity to the Rns binding site consensus sequence TATTTTTTTATC [21]. Since Rns binding sites are not symmetrical, the conserved sequences are either on the coding or noncoding strand of each promoter (Figure 2).

**Figure 3:**
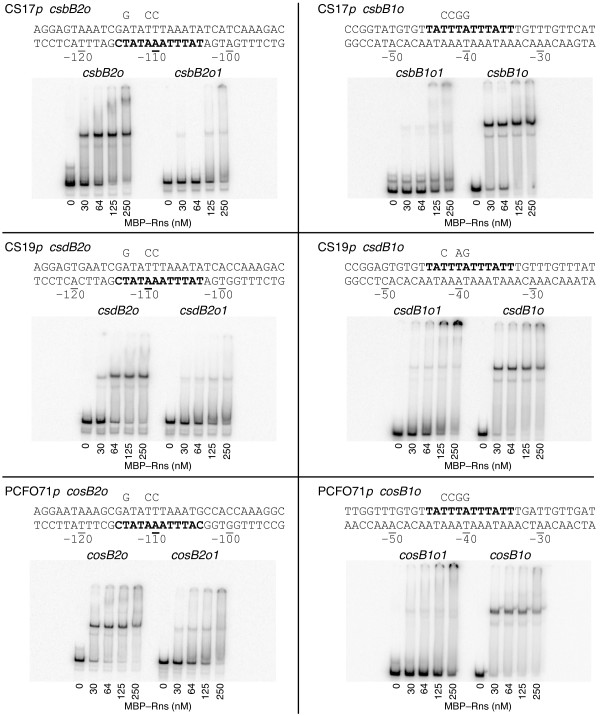
**Gel mobility assays of wild–type and mutagenized Rns binding sites**. The sequence of each binding site is shown with numbering relative to the Rns–dependent transcription start site of each promoter. For the gel mobility assays additional flanking sequences were included with DNA fragments ranging in size from 148 to 257 bp. Nucleotides within the conserved core of each binding site are shown in bold. Point mutations within each binding site are shown above each sequence. Mutagenized binding sites are designated with allele numbers at the end of each site's name. Since each DNA fragment was produced by PCR, primer annealing to sequences with partial homology sometimes produced faint secondary bands as evident in lanes without MBP–Rns.

Since DNase I footprinting identified two Rns binding sites at each fimbrial promoter, each site was subjected to oligonucleotide directed mutagenesis to determine its function with regards to promoter activation. Prior to in vivo analyses, we used gel mobility assays to determine if the point mutations reduced or abolished Rns binding in vitro. We found that the ability of MBP-Rns to alter the mobility of DNA fragments carrying mutagenized sites was substantially reduced compared to wild-type binding sites (Figure 3). This was expected because three or four nucleotides within the conserved core of each site were changed. In addition to large nonspecific protein-DNA complexes trapped in the wells of most gels at high concentrations of MBP-Rns, we also observed a low mobility complex with the wild-type *csbB2o *binding site. This suggests the presence of an additional low affinity binding site that was not observed by DNase I footprinting. This site is likely a pseudo binding site and is probably not relevant in vivo because high concentrations of protein were required for binding.

**Figure 4:**
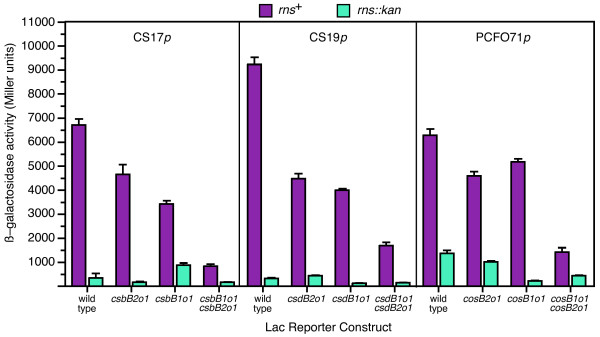
**Function of Rns binding sites in vivo**. Rns–dependent and Rns–independent expression of β-galactosidase from reporter constructs integrated into the chromosome of K-12 strain MC4100. Cells were harvested from overnight cultures with an optical absorbance between 0.7 and 0.8 at 580 nm. Mutagenized binding sites are designated with allele numbers at the end of each site's name. *n *≥ 3

After gel mobility assays, we evaluated the function of each wild-type and mutagenized binding site in vivo by β-galactosidase assays. Overall levels of enzymatic activity were higher in stationary phase (Figure 4) than late log phase (Table 1) because as explained above, β-galactosidase is a stable enzyme that accumulates over time. For each promoter, we observed that mutations within either binding site reduced Rns-dependent expression of the enzyme (Figure 4). However, the most dramatic reductions were observed when both binding sites were simultaneously mutagenized. Taken together, these results demonstrate that the CS17, CS19, and PCFO71 fimbrial promoters each carry two functional binding sites that are required for full Rns-dependent activation.

## Discussion

In this study we have shown that CS17, CS19, and PCFO71 fimbriae are positively regulated by Rns. Although the three promoters expressed different absolute levels of β-galactosidase, the relative activation of each was similar (Table 1). Each fimbrial promoter contains a Rns binding site adjacent to its predicted -35 hexamer and a second binding site centered at -109.5 or -108.5. Point mutations within these binding sites demonstrated that both sites are essential for full Rns-dependent activation of each fimbrial promoter. Moreover, the molecules bound to these sites apparently function independently of one another, with each having an additive rather than synergistic effect upon fimbrial promoter activation. This suggests that Rns's activation of fimbrial promoters is mechanistically distinct from its positive autoregulation because a previous study has shown that positive autoregulation requires synergy between two binding sites [22].

In terms of Rns binding site positions and number; the CS17, CS19, and PCFO71 promoters are similar to the previously described CS1 fimbrial promoter [17]. The four fimbrial promoters also conserve the orientation of each site with the closest match to the consensus binding sequence on the coding strand for promoter proximal sites and the noncoding strand for promoter distal sites. In each case, the center-to-center distance between each pair of binding sites is exactly 70 base pairs. Assuming these sequences are entirely B-form DNA in solution with 10.5 base pairs per helical turn, this distance of 6.7 helical turns would place the activator at the promoter-distal site on the opposite face of the DNA helix relative to the activator bound to the promoter-proximal site. Since we have shown that the promoter distal sites are required for full activation of each promoter, these observations suggest that Rns must reach around the DNA helix when bound at the distal sites in order to interact with RNA polymerase. This reaching may be accomplished by a flexible linker, which we have recently identified (Bodero and Munson, unpublished data) that joins the amino-terminal domain of Rns to its carboxy-terminal DNA binding domain. We have also recently identified residues near the amino-terminus of Rns that are involved in promoter activation [23].

Based on homology between fimbrial proteins; CS1, CS17, CS19, and PCFO71 have been classified as subclass 5b adhesive fimbriae. Two other fimbrial subclasses have been described. Subclass 5a contains CFA/I, CS4, and CS14 fimbriae, whereas subclass 5c contains only CS2 fimbriae. Although only the CFA/I promoter has been characterized in detail [21,23], all ETEC 5a and 5c fimbriae are regulated by Rns, or its functional homolog CfaD [15,24-26]. Thus, with the inclusion of this study, it is now apparent that all known class 5 fimbriae are regulated by Rns/CfaD in ETEC.

Class 5 fimbriae are not the only fimbriae regulated by Rns. Rns also is required for the expression of class 2 CS3 fimbriae which belong to the classical, as opposed to the alternate, chaperone-usher pathway group [27]. CS20 fimbriae are probably also regulated by Rns/CfaD because a fimbrial subunit was hyper-expressed when *cfaD *was provided in trans [28]. This indicates that nearly half of all known ETEC fimbriae are positively regulated by Rns/CfaD. This may actually be an underestimate because we cannot determine if the expression of several fimbriae is Rns-dependent or Rns-independent from the available literature. Nevertheless, the inactivation of Rns would render a significant number of ETEC strains avirulent because Rns controls the expression of some of the most frequently identified fimbriae [6]. In addition, we have shown in this study that Rns activates the expression of CS17, CS19, and PCFO71 fimbriae which are not included in any of the ETEC vaccines currently under development [7,29].

Given the challenges and uncertainty facing ETEC vaccines containing fimbrial antigens [7], it would be prudent to consider other approaches to prevent ETEC infections. A potentially broadly effective approach would be to inactivate Rns since this would eliminate the expression of almost 50% of all known ETEC adhesive fimbriae. Since Rns is a cytoplasmic protein, it is obviously not a suitable antigen for vaccine development. However, it may be possible to identify small molecules that inhibit the activity of Rns by high-throughput screening of chemical libraries. A similar strategy has identified small molecular inhibitors of a *Vibrio cholerae *virulence regulator [30]. If inhibitors of Rns can be identified, they could serve as lead compounds for the development of novel drugs that prevent or ameliorate most ETEC infections.

## Conclusion

This study demonstrates that Rns is required for the expression of all known subclass 5b fimbriae of ETEC. Other studies have shown that Rns is required for the expression of more distally related fimbriae including, but not limited to, those within subclasses 5a and 5c. Taken together, these studies suggest that the inactivation or inhibition of Rns could be an effective strategy to prevent ETEC infections.

## Methods

### Plasmids and strains

Bacterial strains are described in Table 2. The CS17 fimbrial promoter was amplified from strain WS6788A with primers SN419-Bam and SN552-Eco. Primer sequences are listed in Table 3. The CS19 promoter was amplified from strain WS0115A with primers SN421-Bam and SN552-Eco. The PCFO71 promoter was amplified from strain WS2173A with primers SN530-Bam and SN553-Eco. The 0.7 kb PCR products were digested with BamHI and EcoRI, then ligated into the same sites of pHKLac1 to construct pCS17Lac2, pCS19Lac2, and pCFO71Lac2. Plasmid pHKLac1 carries *lacZYA *flanked by transcriptional terminators, *aadA*, *attP*_HK022 _and the *pir*-dependent γ origin of replication from R6K [20]. It is a derivative of pAH144 (GenBank:AY048731) with the addition of a 5.5 kb BamHI-MfeI fragment carrying *lacZYA *from pRS550 [31,32].

**Table 2:** Bacterial strains

Strain	Description	Source or reference
KS1000	K-12; F' *lacIq lac*+*pro*+/*ara*Δ(*lac-pro*)Δ*prc::kan eda51::Tn10 gyrA rpoB thi-1 argI*(am)	New England Biolabs
MC4100	K-12; F- *araD139 *Δ (*argF-lac*)U169 *rpsL150 relA1 deoC1 ptsF25 rbsR*	[36]
WS6788A	ETEC_human_; CS17^+ ^LT^+ ^O8:H9	[18]
WS0115A	ETEC_human_; CS19^+ ^LT^+ ^ST^+ ^O114:H^---^	[18]
WS2173A	ETEC_human_; PCFO71^+ ^LT^+ ^O71:H4	[18]
GPM1133	MC4100 *attB*_HK022_::pCS17Lac2 CS17*p*(-134 to +544)::*lacZYA*	This study
GPM1155	MC4100 *attB*_HK022_::pCS17Lac4 CS17*p*(-134 to +544, *csbB2o1*)::*lacZYA*	This study
GPM1141	MC4100 *attB*_HK022_::pCS17Lac3 CS17*p*(-134 to +544, *csbB1o1*)::*lacZYA*	This study
GPM1232	MC4100 *attB*_HK022_::pCS17Lac5 CS17*p*(-134 to +544, *csbB2o1 csbB1o1*)::*lacZYA*	This study
GPM1134	MC4100 *attB*_HK022_::pCS19Lac2 CS19*p*(-133 to +545)::*lacZYA*	This study
GPM1156	MC4100 *attB*_HK022_::pCS19Lac3 CS19*p*(-133 to +545, *csdB2o1*)::*lacZYA*	This study
GPM1142	MC4100 *attB*_HK022_::pCS19Lac4 CS19*p*(-133 to +545, *csdB1o1*)::*lacZYA*	This study
GPM1234	MC4100 *attB*_HK022_::pCS19Lac5 CS19*p*(-133 to +545, *csdB2o1 csdB1o1*)::*lacZYA*	This study
GPM1131	MC4100 *attB*_HK022_::pCFO71Lac2 PCFO71*p*(-208 to +541)::*lacZYA*	This study
GPM1157	MC4100 *attB*_HK022_::pCFO71Lac4 PCFO71*p*(-208 to +541, *cosB2o1*)::*lacZYA*	This study
GPM1158	MC4100 *attB*_HK022_::pCFO71Lac3 PCFO71*p*(-208 to +541, *cosB1o1*)::*lacZYA*	This study
GPM1235	MC4100 *attB*_HK022_::pCFO71Lac5 PCFO71*p*(-208 to +541, *cosB2o1 cosB1o1*)::*lacZYA*	This study

Numbering of promoter sequences is relative to their Rns–dependent transcription start sites.

**Table 3:** Oligonucleotides

NAME	SEQUENCE
SN419-Bam	GCGGGATCCGACGTCGCAGGAGTAAATCG

SN420-Eco	CGCGAATTCGCCTTCGTAACAAAGGG

SN421-Bam	GCGGGATCCGACGTCGCAGGAGTGAATCG

SN530-Bam	GAGGATCCAAGAACTCGGGGCAGTTC

SN531-Eco	GTGAATTCTTTTGATATTGGATAG

SN552-Eco	GCTGAATTCATAATACCAAGTCTTACATTAC

SN553-Eco	GCAGAATTCCGACCCGCACATTTCCTGTG

SN560-AgeI	GTTACCGGTTTATTTGATTGTTGATTG

SN561-AgeI	AAACCGGTAACACAAACCAACCATCACAAC

SN562-AgeI	GTTACCGGTTTATTTGTTTGTTCATTGTT

SN563-AgeI	AAACCGGTAACACATACCGGCCATTACAAC

SN568-KpnI	GTCGGTACCTAAATATCATCAAAGACAAGTG

SN569-KpnI	TTAGGTACCGATTTACTCCTGCGACGTC

SN570-KpnI	GGCGGTACCTAAATGCCACCAAAGGCAAGC

SN571-KpnI	TTAGGTACCGCTTTATTCCTGCGACATCA

SN572-SpeI	TTACTAGTTTATTTGTTTGTTTATTATTTT

SN573-SpeI	AAACTAGTAACACACTCCGGCCATTAC

SN574-KpnI	TCGGTACCTAAATATCACCAAAGAC

SN575-KpnI	TAGGTACCGATTCACTCCTGCGACGTC

Underlined nucleotides denote primer-template mismatches.

For the purpose of mutagenesis, each reporter plasmid was digested with PvuII then ligated to excise 5 kb of *lacZYA*. The resulting plasmids (pCS17c, pCS19c, and pCFO71c) were then subjected to oligonucleotide directed mutagenesis to create point mutations in Rns binding sites. The point mutations were designed so that they also generate unique restriction sites. Plasmid pCS17c was subjected to inverse PCR with primers SN562-AgeI and SN563-AgeI or SN568-KpnI and SN569-KpnI. Plasmid pCS19c was subjected to inverse PCR with primers SN572-SpeI and SN573-SpeI or SN574-KpnI and SN575-KpnI. Plasmid pCFO71c was subjected to inverse PCR with primers SN560-AgeI and SN561-AgeI or SN570-KpnI and SN571-KpnI. The 3.6 kb PCR products were then digested with AgeI, KpnI, or SpeI then ligated to yield pCS17cAge, pCS17cKpn, pCS19cSpe, pCS19cKpn, pCFO71cAge, and pCFO71cKpn. The mutagenized promoter fragments were then cloned into pHKLac1 as 0.7 kb BamHI-EcoRI fragments. Plasmids containing double mutations, where both Rns-binding sites are mutagenized, were generated by subjecting plasmids pCS17Lac3, pCS19Lac4, and pCFO71Lac3 to an inverse PCR reaction with primers SN568-KpnI and SN569-KpnI, SN574-KpnI and SN575-KpnI, and SN570-KpnI and SN571-KpnI, respectively. The PCR products were then digested with KpnI and recircularized.

Plasmid pCS17Lac3 carries CS17*p *with the unique AgeI restriction site in the Rns promoter proximal binding site *csbB1o*. pCS17Lac4 carries CS17*p *with the unique KpnI site in the Rns promoter distal binding site *csbB2o*. pCS17Lac5 carries both unique restriction sites. Plasmids pCS19Lac3 and pCS19Lac4 carry CS19*p *with a unique KpnI site in the promoter distal Rns binding site *csdB2o *or a SpeI site in the promoter proximal binding site *csdB1o*, respectively. pCS19Lac5 carries both mutations. Plasmids pCFO71Lac3 and pCFO71Lac4 carry CFO71*p *with a unique AgeI site in the promoter proximal Rns binding site *cosB1o *or a KpnI site in the promoter distal binding site *cosB2o*, respectively. pCFO71Lac5 carries both mutations. Reporter plasmids were integrated into the chromosomal *attB*_HK022 _site of MC4100 by Int_HK022 _dependent, site-specific recombination [31]. Strains with single plasmid integrants were identified by colony PCR [31] and used for enzymatic assays.

Plasmid pRARE2 (EMD Biosciences) provides seven rare tRNAs to supplement the rare codon usage of *rns*. Plasmid pMBPRns1 expresses MBP-Rns from an isopropyl-β-D-1-thiogalactopyranoside (IPTG)-inducible *tac *promoter [20]. Plasmid pGPMRns is a derivative of pNEB193 (New England Biolabs) and expresses Rns from *lacp *[20]. Plasmid pGPMRns<Tn>2 carries *rns::kan *and was produced by transposon mutagenesis of pGPMRns.

### Enzymatic assays

Reporter strains were transformed with pGPMRns (*rns*+ *bla*) or pGPMRns<Tn>2 (*rns::kan bla*) and grown aerobically at 37°C in Luria-Bertani (LB) medium with 100 μg/ml ampicillin. Cells were harvested, lysed and assayed for β-galactosidase activity as previously described [33]. Stationary phase cells were harvested from overnight cultures with an optical absorbance between 0.7 and 0.8 at 580 nm. Late log phase cells were harvested 3 to 4 hours after inoculation when the optical absorbance of the culture reached 0.4 to 0.6 at 580 nm.

### Purification of MBP-Rns

Strain KS1000/pRare2/pMBPRns1 was grown aerobically at 37°C in LB medium containing 0.2% wt/vol glucose, 30 μg/ml chloramphenicol, and 100 μg/ml ampicillin. During mid-log phase the incubation temperature was lowered to 30°C then IPTG was added to a final concentration of 300 μM to induce the expression of MBP-Rns. After several hours of induction, cells were harvested at 4°C and concentrated ≥ 100 fold in ice-cold lysis buffer (10 mM TrisCl [pH 7.6], 200 mM NaCl, 1 mM EDTA, 0.5 mM CaCl_2_, 10 mM β-mercaptoethanol, 100 μg/ml DNase I). A French press was used to mechanically lyse the cells. The lysate was clarified by high-speed centrifugation to pellet insoluble material. The supernatant was passed through an amylose column that was then washed with several column volumes of buffer A (10 mM TrisCl [pH 7.6], 200 mM NaCl, 1 mM EDTA, 15% vol/vol glycerol, and 10 mM β-mercaptoethanol). The fusion protein was eluted from the column with buffer B (buffer A with 10 mM maltose). The eluted protein was stored at -80°C in 10 mM TrisCl [pH 7.6], 50 mM KCl, 1 mM β-mercaptoethanol, 30% vol/vol glycerol after buffer exchange. The concentration of MBP-Rns was determined by the Bradford method relative to a standard curve of bovine serum albumin [34].

### DNase I footprinting

MBP-Rns was equilibrated with ^32^P-end-labeled promoter DNA at 37°C in 10 mM TrisCl [pH 7.6], 50 mM KCl, 1 mM DTT, 0.4 mM MgCl_2_, 0.2 mM CaCl_2_, 2 ng/μl polydI-dC, 10 μg/ml bovine serum albumin. After equilibration, DNase I was added to a final concentration of 100 ng/μl for 1 min. at 37°C. The enzymatic reaction was quenched by addition of 10 volumes of 570 mM NH_4_OAc, 50 μg/ml tRNA, 80% vol/vol ethanol. The solution was vortexed then placed on dry ice to precipitate DNA. The DNA was pelleted, washed with 70% vol/vol ethanol, dried, then resuspended in 4 μl 80% vol/vol formamide, 50 mM Tris-Borate [pH 8.3], 1 mM EDTA, 0.1% wt/vol xylene cyanol and bromophenol blue. The samples were heat denatured and separated on sequencing gels. Dried gels were visualized by exposure to phosphorimager plates. The Maxam-Gilbert method was used to generate sequence ladders [35].

### Gel mobility assays

MBP-Rns was equilibrated with ^32^P-labeled CS17, CS19, or PCFO71 promoter fragments in 10 mM TrisCl (pH 7.6), 50 mM KCl, 1 mM DTT, 2 ng/ul poly (dI-dC), 0.1 mg/ml bovine serum albumin, and 6% (vol/vol) glycerol at 37°C for 20 minutes. After equilibration, the reactions were loaded onto non-denaturing 5% acrylamide gels with TAE (40 mM Tris-acetate, 1 mM EDTA [pH 8.5]) as the gel and running buffer. The gels were run at 150 volts for 2 hours, dried, and visualized by exposure to phosphorimager plates.

### Transcription start site mapping

Cells were harvested from cultures after they reached an optical absorbance of 1.0 at 595 nm. Total RNA was isolated from reporter strains GPM1133 (CS17*p::lacZYA*), GPM1134 (CS19*p::lacZYA*), and GPM1131 (PCFO71*p::lacZYA*) transformed with pGPMRns (*rns*+ *bla*) or pGPMRns<Tn>2 (*rns::kan bla*) as previously described [20]. Subsequently, 2.4 picomoles of a ^32^P-end-labeled oligonucleotide (SN420-Eco for CS17*p *and CS19*p*; SN531-Eco for PCFO71*p*) was combined with 54–169 μg of total RNA and 0.8 mM dNTPs. The solution was heated to 65°C for 5 minutes then cooled on ice for 2 minutes to anneal the primer, which was then extended with SuperScript™ III Reverse Transcriptase according to the supplier's protocol (Invitrogen). Aliquots were separated on DNA sequencing gels alongside Maxam-Gilbert sequencing ladders after heat denaturation.

## Authors' contributions

MDB carried out DNA binding studies, transcription start site mapping, molecular cloning, mutagenesis, enzymatic assays, and assisted draft the manuscript. EAH carried out molecular cloning, mutagenesis, and enzymatic assays. The study was conceived, designed, and coordinated by GPM, who also drafted the manuscript and purified protein. All authors read and approved the final manuscript.
